# The role of microRNAs in the regulation of cancer stem cells

**DOI:** 10.3389/fgene.2013.00295

**Published:** 2014-01-03

**Authors:** Ryou-u Takahashi, Hiroaki Miyazaki, Takahiro Ochiya

**Affiliations:** ^1^Division of Molecular and Cellular Medicine, National Cancer Center Research InstituteTokyo, Japan; ^2^Department of Oral and Maxillofacial Surgery, Showa University School of DentistryTokyo, Japan

**Keywords:** microRNA, cancer stem cells (CSCs), tumor initiation, therapy resistance, metastasis

## Abstract

Cancer stem cells (CSCs) have been reported in many human tumors and are proposed to drive tumor initiation and progression. CSCs share a variety of biological properties with normal somatic stem cells such as the capacity for self-renewal, the propagation of differentiated progeny, and the expression of specific cell surface markers and stem cell genes. However, CSCs differ from normal stem cells in their chemoresistance and tumorigenic and metastatic activities. Despite their potential clinical importance, the regulation of CSCs at the molecular level is not well-understood. MicroRNAs (miRNAs) are a class of endogenous non-coding RNAs that play an important role in the regulation of several cellular, physiological, and developmental processes. Aberrant miRNA expression is associated with many human diseases including cancer. miRNAs have been implicated in the regulation of CSC properties; therefore, a better understanding of the modulation of CSC gene expression by miRNAs could aid the identification of promising biomarkers and therapeutic targets. In the present review, we summarize the major findings on the regulation of CSCs by miRNAs and discuss recent advances that have improved our understanding of the regulation of CSCs by miRNA networks and may lead to the development of miRNA therapeutics specifically targeting CSCs.

## Background

The CSC theory, which is based on the concept that cancer might arise from a rare population of cells with stem cell properties, was proposed approximately 150 years ago (Cohnheim, 1875; Wicha et al., 2006). Recent technological developments (flow cytometry analysis and cell sorting) and the establishment of new animal models have provided evidence supporting the CSC theory. Moreover, CSCs are resistant to conventional treatments and are therefore not only of academic interest, but may also be an important consideration in clinical practice. Therefore, a better understanding of the characteristics of CSCs and the identification of therapeutic agents capable of targeting the CSC population are critical issues. Cancer researchers have investigated protein-coding genes and products, including surface markers that are involved in the self-renewal and asymmetric cell division of CSCs. Recently, in addition to alterations in protein-coding genes, abnormalities in non-coding RNAs [miRNAs and long intergenic non-coding RNAs] have been observed in various types of cancers and have been shown to play important roles in the regulation of CSC properties such as asymmetric cell division, tumorigenicity, and drug resistance. In the present review, we discuss the general features of CSCs and the role of miRNAs in the regulation of CSC properties, and summarize the current therapeutic strategies targeting miRNAs for CSC therapy.

## Biogenesis and functions of miRNAs

**Figure 1:**
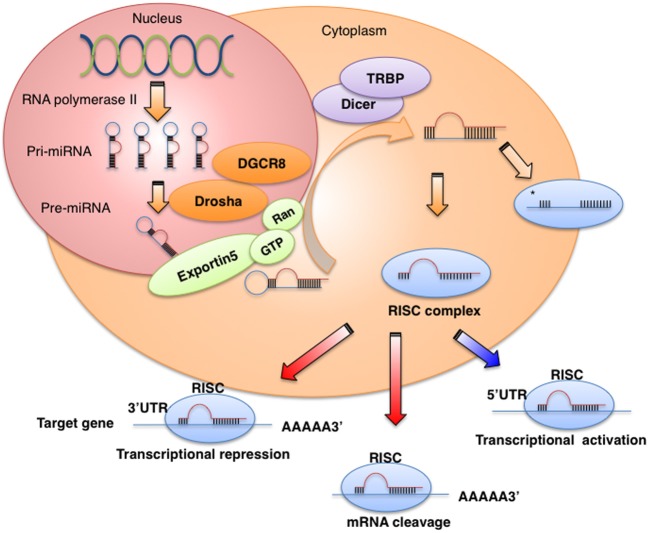
**miRNA biogenesis and function**. miRNAs are transcribed by RNA polymerase II or III as pri-miRNA, and are processed in the nucleus by Drosha-DGCR8 into pre-miRNAs. The product of pri-miRNA cleavage, the pre-miRNA, is exported to the cytoplasm by exportin-5 and further cleaved in a complex composed of Dicer and TRBP. The functional strand of mature miRNA is incorporated into the RNA-induced silencing complex (RISC), which contains GW182 and Argonaute protein. As a part of this complex, the mature miRNA regulates gene expression by binding to partially complementary sequences in the 3′UTRs of target mRNAs, leading to mRNA degradation or translation inhibition.

miRNAs are 21–25 nucleotides long, non-coding RNAs that regulate gene expression at the post-transcriptional level by binding to the 3′-untranslated regions (3′UTRs) or the open reading frames of target genes, leading to the degradation of target mRNAs or repression of mRNA translation (Iorio and Croce, 2012). miRNAs are transcribed for the most part by RNA polymerase II as long primary transcripts characterized by hairpin structures (pri-miRNA), and are processed in the nucleus by RNase III Drosha into 70–100 nucleotide long precursor miRNAs (pre-miRNAs) in combination with cofactors such as DGCR8, an evolutionarily conserved protein that interacts with proline-rich peptides through its WW domain (Gregory et al., 2004; Lee et al., 2004) (Figure 1). *DGCR8* is located on chromosome region 22q11.2, whose heterozygous deletion results in the most common human genetic deletion syndrome, known as DiGeorge syndrome. The clinical symptoms of the disease are highly variable and in approximately 75% of patients, congenital heart defects are observed (Shiohama et al., 2003; Yamagishi and Srivastava, 2003). The product of pri-miRNA cleavage, the pre-miRNA, is exported to the cytoplasm by exportin-5, a member of the Ran-dependent nuclear transport receptor family (Lee et al., 2004) and further cleaved in a complex composed of RNase III Dicer and the transactivating response RNA- binding protein (TRBP) into a miRNA:miRNA^*^ complex. While one of the two strands is selected as a guide strand, the complementary strand (miRNA^*^) is usually degraded (Iorio and Croce, 2012). miRNA^*^ was originally considered to have no function and to be degraded; however, recent evidence suggests that it can be used as a functional strand and may play significant biological roles (Uchino et al., 2013; Yang et al., 2013).

The mature miRNA is incorporated into a complex known as the RNA-induced silencing complex (RISC), which contains the GW182 and Argonaute proteins. As a part of this complex, the mature miRNA regulates gene expression by binding to partially complementary sequences in the 3′UTRs of target mRNAs, leading to mRNA degradation or translation inhibition (Iorio and Croce, 2012). Several studies have reported that miRNAs also bind to the 5′UTR or the open reading frame (Orom et al., 2008; Mandke et al., 2012) and can promote the translation of their target genes under growth arrest conditions (Vasudevan et al., 2007). Recently, Nishi et al. showed that TNRC6A, a human GW182 paralog, shuttles Ago2 into the nucleus and the colocalization of Ago2-TNRC6A with miRNAs mediates gene silencing (Nishi et al., 2013).

## MicroRNAs regulate pluripotency and differentiation

The discovery of two miRNAs, lin-4 and let-7, in *Caenorhabditis elegans* suggested that miRNAs are important regulators of embryonic development and stem cell functions in mammals (Lee et al., 1993; Pasquinelli et al., 2000; Reinhart et al., 2000). The function of miRNAs in mouse and human embryonic stem cells (ESCs) has been investigated using cells lacking Dicer1 and DGCR8, which are critical for miRNA biogenesis. Deletion of Dicer1 leads to embryonic lethality in mice (Bernstein et al., 2003) and DGCR8-deficient mouse ESCs show alterations in the regulation of the cell cycle and differentiation that are associated with failure to silence stemness markers, such as *Oct4, Rex1, Sox2*, and *Nanog*, as well as delayed expression of differentiation markers (Wang et al., 2007).

In a comparative transcriptome analysis, Dicer1-deficient mouse ESCs lacking miRNAs showed a significant increase in transcripts containing a GCACUU motif in the 3′UTR (Sinkkonen et al., 2008). This sequence is complementary to the AAGUGC seed sequence of the miR-290-295 cluster (miR-290, miR-291a, miR-292, miR-291b, miR-294, and miR-295) and the miR-302/367 cluster (miR-302a, miR-302b, miR-302c, miR-302d, and miR-367) in mouse ESCs. Using a similar approach, novel stem cell-specific miRNAs were initially identified in human ESCs. These miRNAs include two clusters: miR-302/367 and the miR-371 cluster (miR-372 and miR-373). The expression of the miR-371 cluster is downregulated before that of the miR-302/367 cluster, suggesting a temporal hierarchy in the duration of specific miRNA activity (Stadler et al., 2010; Kim et al., 2011). Members of the miR-302 family rescue the proliferation defects of DGCR8-mutant mouse ESCs (Wang et al., 2008) and reprogram human skin cancer cells into a pluripotent ESC-like state (Lin et al., 2008).

**Figure 2:**
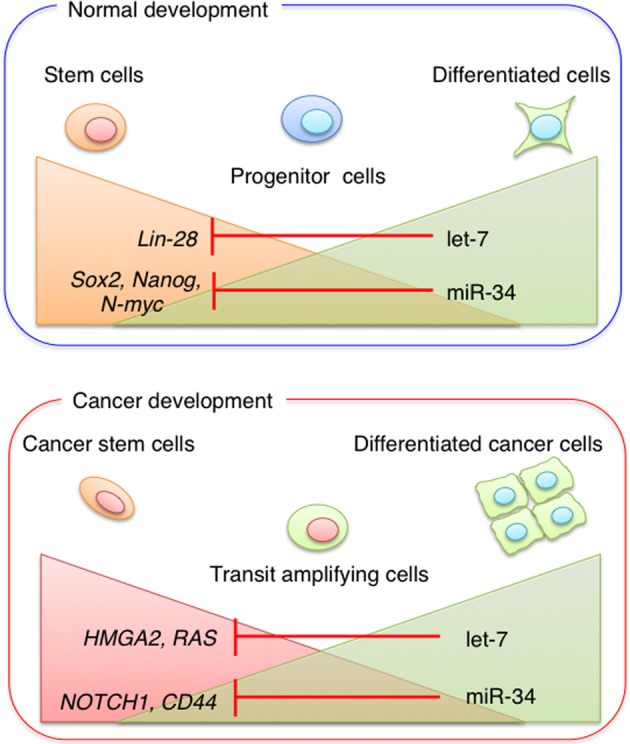
**miRNA in stem cells and cancer stem cells**. Stem cell-specific miRNAs play important roles in tumor initiation and development. During normal development, pluripotent stem cells become more restricted to specific cell lineages. Progenitor cells are committed to generating different cell types, whereas fully differentiated cells have a low potential for self-renewal. The expression levels of miR-34 and let-7 family members increase during differentiation. During cancer development, CSC properties are regulated by the balance between miRNA expression and the expression of miRNA target genes.

The Let-7 family is another critical regulator of ESC differentiation. Mature let-7 family members are essentially absent in ESCs and accumulate only upon ESC differentiation (Viswanathan et al., 2008). Melton et al. reported that whereas transfection of let-7c into wild-type cells had no effect on the expression of pluripotency genes, let-7c rescued the differentiation defect in DGCR8^−/−^ cells by downregulating *Oct4, Sox2*, and *Nanog* (Melton et al., 2010). Lin-28, a marker of undifferentiated ESCs, is also used to induce pluripotent stem cells (Yu et al., 2007b). A negative feedback loop between Lin-28 and let-7 family members precisely controls the levels of these miRNAs. Although Lin-28 regulates the expression of let-7 miRNAs by binding to the precursors and blocking their maturation, the let-7 family is highly expressed and targets Lin-28 mRNA in mouse differentiated cells and embryonic carcinoma cells (Yu et al., 2007b) (Figure 2). Members of the miR-34 family of miRNAs are direct targets of p53 and function as tumor suppressors, inhibiting reprogramming through the repression of pluripotency genes such as *Nanog, Sox2*, and *N-myc* (Choi et al., 2011) (Figure 2). Since the cell cycle regulator p21 also represses reprogramming efficiency, these findings suggest that p53 represses pluripotency via two distinct mechanisms. Evidence that let-7 and miR-34 family members are tumor suppressor miRNAs (Takamizawa et al., 2004; Johnson et al., 2005; Tazawa et al., 2007) suggests that stem cell-specific miRNAs play important roles in tumor initiation and development.

## miRNA regulation in cancer

miRNAs play a crucial role in the progression of human cancer, and expression profiling in human malignancies has identified signatures associated with cancer development, progression, and prognosis (Liu et al., 2012; Volinia and Croce, 2013). Chromosomal regions coding for oncogenic miRNAs that are involved in the negative regulation of a tumor suppressor gene can be amplified in association with cancer development. This amplification would result in the upregulation of oncogenic miRNAs and silencing of tumor suppressor genes (He et al., 2005). On the other hand, miRNAs targeting oncogenes are often located in fragile site, where deletions or mutations can occur, leading to the reduction or loss of miRNAs and the overexpression of their target oncogenes. Dysregulation of miRNA expression affects processes associated with cancer progression such as the induction of anti-apoptotic activity, drug resistance, tissue invasion, and metastasis (Cimmino et al., 2005; Tavazoie et al., 2008; To et al., 2008). Recent evidence suggests that miRNAs are involved in tumor initiation through the regulation of CSC properties such as self-renewal ability, tumorigenicity and drug-resistance (Yu et al., 2007a; Shimono et al., 2009; Song et al., 2013a,b).

## CSCs

Accumulating lines of evidence suggest that CSCs share a variety of biological properties with normal somatic stem cells such as the capacity for self-renewal, the propagation of differentiated progenitors, and the expression of specific stem cell genes (Colmont et al., 2012). However, CSCs differ from normal stem cells in their chemoresistance and tumorigenic and metastatic activities (Colmont et al., 2012 and Table 1). In addition, recently glycosylation patterns are found to be different between normal stem cells and CSCs (Karsten and Goletz, 2013). The CSC theory is generally accepted in the field of cancer research, not only in basic research but also with regard to cancer drug discovery.

**Table 1:** **Representative cell surface markers for human CSCs**.

**Cancer type**	**CSC marker**	**References**
AML	CD34^+^/CD38^−^	Bonnet and Dick, 1997
Breast	CD44^+^/CD24^−/^low	Al-Hajj et al., 2003
	ALDH1	Ginestier et al., 2007
Glioma	CD133	Singh et al., 2003, 2004
Colon	CD133	O'brien et al., 2007; Ricci-Vitiani et al., 2007
	CD44/EpCAM/CD166	Dalerba et al., 2007
*Metastatic* Colon	CD133^+^/CD26^+^	Pang et al., 2010
Melanoma	CD20	Fang et al., 2005
	CD271	Boiko et al., 2010
Pancreatic	ESA/CD44/CD24	Hermann et al., 2007
*Metastatic* Pancreatic	CD133/CXCR4	Li et al., 2007a
Prostate	CD44/a2β1/CD133	Collins et al., 2005
Lung	CD133	Eramo et al., 2008
Hepatic	EpCAM/AFP	Yamashita et al., 2010
Gastric	CD44	Takaishi et al., 2009

*AML, acute myelogenous leukemia; ALDH, aldehyde dehydrogenase; EpCAM, epithelial cell adhesion molecule; CXCR4, CXC chemokine receptor 4; AFP, alpha-fetoprotein*.

Normal stem cells and CSCs act via common signaling pathways that regulate self-renewal activity, including Wnt, Notch, and Sonic Hedgehog, and dysregulation of these pathways plays a role in tumor initiation and development (Reya et al., 2001). Jamieson et al. showed that aberrations in the Wnt/β-catenin pathway enhance self-renewal activity during leukemia stem cell propagation (Jamieson et al., 2004). Korkaya et al. reported that the Wnt/β-catenin pathway is involved in the regulation of normal and malignant mammary stem/progenitor cell populations (Korkaya et al., 2009). Several studies have shown that the Notch pathway is activated in breast, glioblastoma, and colon CSCs (Hoey et al., 2009; Taketo, 2011). Alterations in Hedgehog signaling have been reported in colon, breast, and glioblastoma CSCs (Liu et al., 2006; Varnat et al., 2009; Takezaki et al., 2011).

The development of fluorescent antibodies, flow cytometry, and cell sorting techniques enabled the identification of cell populations possessing CSC properties. Furthermore, the development of severely immunodeficient mouse strains facilitated the evaluation of tumor formation ability. These methods have enabled the identification and isolation of CSCs from various cancers (Bonnet and Dick, 1997; Al-Hajj et al., 2003; Collins et al., 2005; Fang et al., 2005; Ginestier et al., 2007; Hermann et al., 2007; Li et al., 2007a; Eramo et al., 2008; Takaishi et al., 2009; Boiko et al., 2010; Pang et al., 2010; Yamashita et al., 2010) (Table 1). In this review, we discuss the major findings of recent studies highlighting the roles of certain “CSC-specific” miRNAs in representative cancer types (Table 2). From these discussions, we present an emerging theme that several miRNAs may exert a functional role in the regulation of the key biological properties of CSCs.

**Table 2:** **The regulatory roles of miRNAs in CSCs**.

**Cancer Type**	**miRNA**	**Target gene**	**Role of miRNA in CSC properties**	**References**
Leukemia (AML and MDS)	miR-22	*TET2*	Promotion of self-renewal	Song et al., 2013a
Breast	Let-7	*RAS and HMGA2*	Inhibition of self-renewal and de-differentiation	Yu et al., 2007a
	miR-200 family	*ZEB1/ZEB2*	Inhibition of EMT	Gregory et al., 2008
		*BMI-1*	Inhibition of self-renewal	Shimono et al., 2009
		*SUZ12*	Inhibition of mammosphere formation	Iliopoulos et al., 2010
	miR-22	*TET* family *(TET1 -3)*	Suppression of miR-200 family expression	Song et al., 2013b
Brain	miR-9/9^*^, miR-17	*CAMTA1*	Promotion of CD133^+^ cell proliferation	Schraivogel et al., 2011
	miR-128	*BMI-1*	Inhibition of self-renewal	Godlewski et al., 2008
	miR-199b-5p	*HES1*	Reduction of the CD133^+^ cell fraction	Garzia et al., 2009
Colon	miR-193	*PLAU and K-RAS*	Inhibition of tumorigenicity and invasiveness	Iliopoulos et al., 2011
	miR-451	*MIF and COX-2*	Inhibition of self-renewal and tumorigenicity	Bitarte et al., 2011
	miR-34a	*NOTCH 1*	Suppression of asymmetric cell division	Bu et al., 2013
Prostate	miR-34a	*CD44*	Inhibition of self-renewal and metastasis	Liu et al., 2011
	miR-320	β-*catenin*	Inhibition of Wnt/β-catenin pathway	Hsieh et al., 2013

*AML, acute myelogenous leukemia; MDS, myelodysplastic syndrome*.

## Leukemia stem cells

Through an integrated approach that combined miRNA expression analysis and bioinformatic prediction of mRNA targets, distinct miRNA signatures were shown to fine-tune each step of hematopoiesis, including the reconstitution potential of hematopoietic stem cells (Arnold et al., 2011). The miR-17-92 cluster functions as an oncogenic miRNA by enhancing the formation of Myc-driven B-cell lymphomas in a mouse model (He et al., 2005). Single miRNAs function as oncogenes. The overexpression of miR-155 in early B-cells leads to polyclonal expansion of the pro-B-cell compartment (Costinean et al., 2006), and retroviral expression of miR-155 in immature mouse hematopoietic cells resulted in the expansion of granulocyte/monocyte populations displaying pathological features characteristic of myeloid neoplasia without progression to acute myeloid leukemia (AML) (O'connell et al., 2008). Recently, dysregulation of single miRNAs was shown to contribute to hematological malignancies, including AML and myelodysplastic syndrome (Han et al., 2010; Song et al., 2013a). Han et al. reported that miR-29a regulates early hematopoiesis and induces AML by converting myeloid progenitors into self-renewing leukemia stem cells via targeting several tumor suppressors and cell cycle regulators (Han et al., 2010). miR-22-induced inhibition of the ten-eleven-translocation gene 2 (*TET2*) tumor suppressor increased the methylation of TET2 target genes, such as *Aim2, Hal, Igbt2*, and *Sp140*, and resulted in positive effects on hematopoietic stem cell self-renewal and transformation. This has led to the suggestion that mir-22 is associated with myelodysplastic syndrome and hematological malignancies (Song et al., 2013a).

## Breast CSCs

The first solid tumor CSCs were identified in and isolated from breast tumors in 2003 (Al-Hajj et al., 2003). Al-Hajj et al. described a CD44^+^/CD24^−/low^ cell population that had a markedly high tumor-initiating capacity. In 2007, Yu et al. identified let-7 as a master regulator of breast CSC properties (Yu et al., 2007a). In breast CSCs, reduced let-7 expression controls self-renewal and differentiation through *RAS* and *HMGA2*, respectively (Figure 2). Since HMGA2 plays a role in the control of differentiation and proliferation of both human and mouse ESCs (Li et al., 2007b), these findings also suggest that let-7 is involved in the growth and differentiation of ESCs beyond tumorigenesis.

Epithelial-to-mesenchymal transition (EMT) is an evolutionarily conserved process that occurs during embryonic development in many species of mammals (Liu et al., 2006). Since the EMT program is often activated during tumor invasion and metastasis, the genetic controls and biochemical mechanisms underlying the acquisition of invasiveness and the subsequent systemic spread of cancer cells have been areas of intensive research. The EMT phenotype is characterized by the downregulation of epithelial markers such as E-cadherin, the expression of mesenchymal markers such as N-cadherin and vimentin, the loss of cell-cell contact and cell polarity, and the acquisition of cell invasive capabilities. Mani et al. reported that EMT is also associated with the acquisition of CSC properties (Mani et al., 2008). A CD44^+^/CD24^−/low^ cell population purified from cancer tissues shows the features of an EMT phenotype, and human cancer cells induced to undergo EMT exhibit a CD44^+^/CD24^−/low^ antigen phenotype and high tumorigenicity.

Recently, two studies reported the clinical relevance of CSCs in breast cancer specimens (Giordano et al., 2013; Yu et al., 2013). In early breast cancer patients, the presence of CD44^+^/CD24^−/low^ cells in bone marrow was indicative of a poor prognosis (Giordano et al., 2013). Circulating tumor cells (CTCs) in breast cancer patients also showed the EMT phenotype (Yu et al., 2013). Progressive disease patients undergoing therapy had a higher number of mesenchymal marker positive CTCs than epithelial marker positive CTCs. These results suggest that the CSC phenotype is clinically important not only as a therapeutic target but also as a potential biomarker for the prognostic evaluation of patients undergoing cancer treatment.

A molecular link between EMT and the miR-200 family is provided by the zinc-finger E-box-binding homeobox protein encoding genes (*ZEB1/ZEB2*) (Gregory et al., 2008; Park et al., 2008). The miR-200 family consists of five members that are classified into two clusters: miR−200a, miR−200b, and miR−429 on human chromosome 1; and miR−200c and miR−141 on human chromosome 12 (Gregory et al., 2008). Expression of the miR-200 family strongly inhibits the EMT phenotype induced by TGF-β, and a reciprocal feedback loop between the miR-200 family and the ZEB family of transcription factors tightly regulates both EMT and mesenchymal-to-epithelial transition (Burk et al., 2008). MiR-200 family members are downregulated in normal human and mouse mammary stem cells and breast CSCs, and miR-200c inhibits the formation of mammary ducts from mammary stem cells and tumor formation from breast CSCs (Shimono et al., 2009). Members of the miR-200 family also modulate the self-renewal ability of CSCs by targeting B-lymphoma Mo-MLV insertion region 1 homolog (*BMI-1*) and *SUZ12*, a subunit of a polycomb repressor complex (Iliopoulos et al., 2010). BMI-1 regulates the self-renewal and differentiation of several types of stem cells, including hematopoietic, brain, and mammary stem cells (Molofsky et al., 2003; Park et al., 2003; Pietersen et al., 2008). Therefore, modulation of the activity of the miR-200 family using conventional therapy could be a promising approach to improve the effectiveness of breast cancer treatments.

Normal human and mouse mammary stem cells can be isolated and characterized on the basis of their aldehyde dehydrogenase (ALDH) activities (Ginestier et al., 2007). Using ALDH activity, Ibara et al. determined that miR-205 and miR-22 were highly expressed in mouse mammary progenitor cells (Ibarra et al., 2007). MiR-22 was recently shown to be an epigenetic modifier that promotes stemness and metastasis in breast cancer by directly targeting enzymes in the TET family, which regulate DNA demethylation (Song et al., 2013b). The TET family is involved in the demethylation of the miR-200 promoter, and miR-22 promotes CSC properties such as EMT and a metastatic phenotype through the suppression of the miR-200 family. This provides the first evidence that chromatin-remodeling systems with opposing effects on cell fate (self-renewal vs. differentiation) are regulated by opposing sets of miRNAs.

## Brain CSCs

The pentaspan membrane glycoprotein CD133, also known as Prominin-1, was first identified as a marker of hematopoietic stem cells and progenitor cells, and was subsequently used to detect malignancies (Miraglia et al., 1997; Yin et al., 1997). In solid cancers, CD133 was first used to identify CSCs in different types of human brain tumors including glioblastoma, medulloblastoma, and ependymomas (Singh et al., 2003, 2004; Yu et al., 2010). In these studies, patient tumor cells were separated based on the expression of CD133. The CD133^+^ cell population is highly tumorigenic *in vivo*, whereas CD133^−^ cells do not form tumors even at high numbers (Singh et al., 2003, 2004; Yu et al., 2010). CD133^+^ cells are also resistant to radiation and chemotherapy. These findings led to the hypothesis that glioblastomas are maintained by CSCs, and that this treatment-resistant subpopulation is a promising target for effective therapies. CD133 has been instrumental for the identification of CSCs in colorectal (Ricci-Vitiani et al., 2007) and pancreatic (Hermann et al., 2007) carcinomas. CD133 itself is a marker of normal neural stem cells in both humans (Uchida et al., 2000) and mice (Lee et al., 2005).

In cancer cells, the deacetylase HDAC6 directly interacts with and regulates the intracellular localization of CD133 (Mak et al., 2012). CD133 forms a stable protein complex with HDAC6 and β-catenin, which leads to the activation of β-catenin signaling targets in different types of cancer. CD133 is also associated with phosphoinositide 3-kinase (PI3K) 85 kDa regulatory subunit (p85) in glioma stem cells (GSCs) (Wei et al., 2013). The PI3K pathway is a key regulator of tumorigenesis in glioblastoma and other cancers (Godlewski et al., 2010). Therefore, activation of the PI3K/Akt pathway by the physical interaction between CD133 and p85 promotes tumorigenicity in GSCs. The function of CD133 in brain tumors should be fully characterized in the near future, which may shed light on the role of CD133 as a functional marker of GSCs.

Schraivogel et al. reported that miR-9, miR-9^*^ (miR-9/9^*^), miR-17, and miR-106b are highly abundant in the CD133^+^ cell population in glioblastoma cell lines. Among the upregulated miRNAs in the CD133^+^ cell population, inhibition of miR-9/9^*^ or miR-17 leads to reduced neurosphere formation and stimulates cell differentiation. Functional analysis of these miRNAs showed that miR-9/9^*^ and miR-17 target calmodulin-binding transcription activator 1 (*CAMTA1*), a putative transcription factor of the anti-proliferative cardiac hormone natriuretic peptide A (*NPPA*). Clinical studies also demonstrated that *CAMTA1* and *NPPA* expression is correlated with patient survival. These findings could provide a basis for the design of novel treatment strategies for glioblastoma (Schraivogel et al., 2011).

MiR-124 and miR-128 are the most highly expressed miRNAs in the adult brain and are preferentially expressed in neurons (Smirnova et al., 2005). Patients with high-grade glioma show significant downregulation of miR-128 expression. Functional analyses showed that miR-128 expression inhibits glioma cell proliferation *in vitro* and glioma xenograft growth *in vivo* (Godlewski et al., 2008). In addition, miR-128 specifically inhibits the self-renewal capacity of GSCs by directly targeting *BMI-1*, a polycomb family transcriptional repressor required for postnatal maintenance of neural stem cells in the peripheral and central nervous system (Molofsky et al., 2003). Since BMI-1 maintains neural stem cells in an undifferentiated self-renewing state, the regulation of *BMI-1* by miR-128 may contribute to normal stem cell regulation.

Another study showed that miR-199b-5p downregulation was associated with metastatic spread in medulloblastoma. In medulloblastoma cells, miR-199b-5p directly targets *HES1*, a transcription factor of the Notch signaling pathway (Garzia et al., 2009). During brain development, Notch functions as a critical regulator of cell fate, by which gliogenesis can only occur when Notch signaling specifically represses the neuronal pathway in progenitor cells (Karamboulas and Ailles, 2013). MiR-199b-5p blocks Notch signaling, inhibiting the self-renewal capacity of medulloblastoma cells by reducing the CD133^+^ subpopulation (Garzia et al., 2009). Recently, miR-34a was shown to regulate Notch signaling by targeting *Notch-1* and *Notch-2* in medulloblastoma cells (Li et al., 2009). Therefore, miR-199b-5p and miR-34a are important for the self-renewal potential of GSCs via the Notch signaling pathway.

## Colon CSCs

CD133 was initially used to identify and isolate colon CSCs (O'brien et al., 2007; Ricci-Vitiani et al., 2007), which was followed by the identification of CD44, epithelial surface antigen (EpCAM), and CD166 as alternative colon CSC markers (Dalerba et al., 2007). CD166 is a mesenchymal stem cell marker whose expression is correlated with poor prognosis in colon cancer patients (Weichert et al., 2004). Compared to CD44^−^/EpCAM^low^ cells, CD44^+^/EpCAM^high^ cells from primary tumors show high tumorigenic activity in NOD/SCID mice. Moreover, CD166^+^ cells in the CD44^+^/EpCAM^high^ cell fraction contribute to the tumorigenic activity of colon CSCs. In addition to CD133, CD44, EpCAM, and CD166, the expression of leucine-rich repeat-containing G-protein-coupled receptor 5 (Lgr5) varies among colorectal cancer (CRC) cases and is significantly correlated with lymphatic and vascular invasion, lymph node metastasis, and drug resistance (Vermeulen et al., 2008; Merlos-Suarez et al., 2011; Kobayashi et al., 2012).

Iliopoulos et al. reported that the expression of miR-193a is inversely correlated with *K-RAS* and plasminogen activator urokinase (*PLAU*) expression in human colon adenocarcinomas, and that miR-193 expression inhibits tumorigenicity and invasiveness by directly targeting *K-RAS* and *PLAU*, respectively (Iliopoulos et al., 2011). MiR-451 is another regulator of CSC properties such as self-renewal, tumorigenicity, and drug resistance. In spheroid cell culture, downregulation of miR-451 induces the upregulation of macrophage migration inhibitory factor (MIF) and COX-2, resulting in the acquisition of self-renewal and tumorigenic properties (Bitarte et al., 2011). MIF and Cox-2 are involved in the activation of the Wnt pathway, which is functionally essential for the maintenance of colon CSCs (Vermeulen et al., 2010), suggesting that miR-451 could regulate the properties of colon CSCs by suppressing the Wnt pathway.

Notch signaling is frequently activated in CRCs, and is dysregulated directly by epigenetic and genetic changes and indirectly by synergistic interactions with the Wnt pathway, which is also activated in CRC (Taketo, 2011). Notch signaling promotes the self-renewal activity of intestine and colon stem cells (Taketo, 2011). Therefore, colon CSCs in CRC are thought to arise from, or at least share common properties with, normal colon stem cells (Clevers, 2011; O'brien et al., 2012). Bu et al. reported that miR-34a determines whether colon CSCs undergo symmetric or asymmetric division, and that inhibition of asymmetric cell division suppresses tumorigenicity (Bu et al., 2013). MiR-34a inhibits Notch signaling by directly targeting Notch receptors (Li et al., 2009), suggesting that the upregulation of miR-34a weakens Notch signaling and promotes the generation of daughter cells (non-CSCs), whereas low miR-34a levels promote Notch signaling and lead to the maintenance of CSCs. This study also demonstrated that the expression level of miR-34a correlates more closely with the differentiation of daughter cells than the presence of Numb, which also suppresses Notch signaling by promoting the degradation of membrane-bound Notch and its intracellular domain (Bu et al., 2013).

## Prostate CSCs

In prostate cancer (PCa), α_2_ β_1_ integrin, CD133, and CD44 were initially used to identify and isolate CSCs (Collins et al., 2005; Patrawala et al., 2006, 2007). Patrawala et al. reported that CD44^+^ PCa cells have higher proliferative, tumorigenic, and metastatic potentials than CD44^−^ PCa cells (Patrawala et al., 2006), and showed that androgen receptor (AR)-negative CD44^+^ PCa cells differentiate into AR-positive CD44^−^ PCa cells. Consistent with this report, prostate-specific antigen (PSA)-negative or -low PCa cells that are resistant to androgen ablation have a highly tumorigenic phenotype (Qin et al., 2012). In addition, PSA^−/low^ PCa cells generate PSA^+^ PCa cells through asymmetric cell division, and highly tumorigenic PSA^−/low^ PCa cells are characterized by an ALDH^+^/CD44^+^/α_2_β_1_ integrin^+^ phenotype (Qin et al., 2012).

Liu et al. reported that miR-34a is downregulated in CD44^+^ PCa cells purified from xenografts and primary tumors, and that miR-34a directly regulates the expression of *CD44* at the post-transcriptional level by binding to its 3′UTR (Liu et al., 2011). Expression of miR-34a in CD44^+^ PCa cells inhibits tumor migration and metastasis in a xenograft model (Liu et al., 2011), and miR-34a inhibits Notch and AR signaling in PCa cells (Li et al., 2009; Kashat et al., 2012), suggesting that miR-34a suppresses the self-renewal activity of CSCs in PCa cells.

Another miRNA that regulates CSC properties is miR-320, which acts by directly targeting β-catenin in PCa cells (Hsieh et al., 2013). miR-320 and β-catenin expression is inversely correlated in CD44^+^ PCa cells. Furthermore, gene expression profiling of miR-320-overexpressing PCa cells showed a significant decrease in downstream target genes of the Wnt/β-catenin pathway and CSC markers (Hsieh et al., 2013).

## Therapeutic approaches to target CSCs

The development of therapies against CSCs has resulted in the establishment of a new generation of cancer therapeutics, which is particularly important in the treatment of intractable cancers. Since CSCs are molecularly distinct from non-CSCs and bulk tumor cells, a high-throughput screening approach was used to identify small compounds that eliminate or reduce levels of CSCs (Gupta et al., 2009; Sachlos et al., 2012). Gupta et al. identified salinomycin as a selective inhibitor of breast CSCs (Gupta et al., 2009) by screening a library of 16,000 natural and commercial chemical compounds in a search for small compounds capable of killing breast CSCs. Although the precise molecular mechanisms underlying the elimination of CSCs by salinomycin are not fully understood, several studies have improved our understanding of the mechanisms and pharmacological action of salinomycin in human CSCs (Fuchs et al., 2010; Lu et al., 2011; Tang et al., 2011). Systemic salinomycin therapy induces a marked regression of subcutaneous thoracal metastases of breast cancer, and combination therapy of salinomycin with erlotinib resulted in significant tumor regression in metastatic squamous cell carcinoma (Naujokat and Steinhart, 2012).

High-throughput screening using neoplastic and normal human pluripotent stem cells (hPSC) showed that among 590 compounds, only thioridazine significantly promoted differentiation of neoplastic hPSCs but not of normal hPSCs (Sachlos et al., 2012). Thioridazine acts through dopamine receptors (dopamine receptor1-5) (Seeman and Lee, 1975), indicating that its selective interference with human CSCs is mediated by dopamine receptor antagonism.

The development of therapies against CSCs is challenging because both bulk cancer cells and CSCs must be eliminated. As CSCs are molecularly distinct from bulk tumor cells, they can be targeted by exploiting their molecular differences as described above (Tables 1, 2). One of the most promising approaches is the cell based delivery of miRNAs or miRNA inhibitors. Several studies demonstrated that miRNAs are secreted through “exosomes,” which are small endosome-derived vesicles (30–100 nm) secreted from different cell types, such as dendritic cells, hepatocyte, and tumor cells (Mittelbrunn et al., 2011; Luga et al., 2012; Ramakrishnaiah et al., 2013). The exosome secreted from mesenchymal stem cells (MSC) is selectively transferred to the glioblastoma multiforme (GBM) (Munoz et al., 2013). Since miR-9 is involved in the upregulation of p-glycoprotein, Munoz et al. developed an MSC derived exosome containing anti-miR-9 that efficiently suppressed p-glycoprotein expression in the temozolomide-resistant GBM.

The glycosylation pattern of CSC markers on CSCs is different from normal stem cells (Karsten and Goletz, 2013). Some CSC markers such as CD44 and CD133 are also expressed in normal stem and progenitor cells (Karsten and Goletz, 2013), which might have negative implications for the development of CSC-targeted delivery. This problem could be addressed by the development of liposomes or nanoparticles conjugated to antibodies against CSC specific glycans that permit the selective delivery of CSC suppressive miRNAs or small molecules.

Recent studies have shown that several dietary compounds can directly or indirectly affect the properties of CSCs (Li et al., 2011). Therefore, natural dietary compounds have received increasing attention in cancer chemoprevention, and several natural compounds that induce the elimination or differentiation of breast CSCs have been identified (Kakarala et al., 2010; Li et al., 2010; Hagiwara et al., 2012). Resveratrol is a non-toxic natural product that is found in grapes, berries, peanuts and red wine (Aziz et al., 2003). Nowadays, resveratrol is widely consumed as a nutritional supplement (Prasad, 2012), and its multifaceted biological effects include anti-mutagenic and anti-cancer properties (Prasad, 2012; Patel et al., 2013). Hagiwara et al. found that resveratrol enhances miRNA functions through the upregulation of Ago2 expression, which leads to the suppression of CSC properties (Hagiwara et al., 2012). These results suggest that the identification of non-toxic natural compounds capable of suppressing the properties of CSCs through the regulation of miRNA expression is a promising approach to support conventional chemotherapy.

## Conclusions

Accumulating lines of evidence have shown that the heterogeneity and plasticity of cancer cells is reflected in the transition from a non-CSC to a CSC phenotype. Therefore, clinical oncologists and cancer researchers need to determine which cancer cells have the potential to contribute to tumor initiation and progression, including therapeutic resistance and metastasis. Several studies reviewed here have shown that miRNAs can function as tumor suppressors or oncogenes and play important roles in various aspects of CSC properties. In this regard, miRNAs are considered to be functional markers of CSCs. Therefore, a more detailed understanding of the function of miRNAs in CSC biology may improve cancer treatments and possibly lead to the clinical application of miRNAs in cancer diagnosis, treatment, and prognosis.

### Conflict of interest statement

The authors declare that the research was conducted in the absence of any commercial or financial relationships that could be construed as a potential conflict of interest.
